# Spontaneous Coronary Artery Dissection in Complex Congenital Coronary Anatomy

**DOI:** 10.1016/j.jaccas.2026.107618

**Published:** 2026-03-26

**Authors:** Mario José Recio Ibarz, Amanda Leandro Barros, Elena Rodriguez García, Francisco de la Cuerda Llorente, Flavio Augusto Gasparini Noriega, Iván de María Mier, Alberto García Lledó

**Affiliations:** a Cardiology Department, Hospital Universitario Miguel Servet, Zaragoza, Spain; b Cardiology Department, Hospital Universitario Príncipe de Asturias, Madrid, Spain; c Cardiology Department, Hospital Universitario de León, León, Spain; d Cardiology Department, Hospital Universitario de Bellvitge, Barcelona, Spain

**Keywords:** acute coronary syndrome, coronary angiography, coronary vessel anomaly, dissection

## Abstract

**Background:**

Spontaneous coronary artery dissection is a nonatherosclerotic cause of acute coronary syndrome, predominantly affecting women. Congenital coronary artery anomalies are uncommon but may confer mechanical and hemodynamic vulnerability.

**Case Summary:**

We report the case of a 63-year-old woman presenting with acute coronary syndrome in the setting of a rare congenital anatomy, with origin of all 3 coronary arteries from the right coronary sinus. Angiographic and computed tomography findings were consistent with spontaneous coronary artery dissection.

**Discussion:**

Multimodality imaging was essential for defining coronary anatomy, delineating the extent of dissection, and guiding management. A conservative strategy was adopted, given preserved coronary flow and hemodynamic stability, resulting in favorable clinical evolution.

**Take-Home Message:**

This case represents an exceptionally rare presentation of spontaneous coronary artery dissection in the setting of such complex coronary anatomy and underscores the importance of recognizing these cases by using multimodality imaging to avoid potentially harmful interventions.

## Visual Summary

**Figure:**
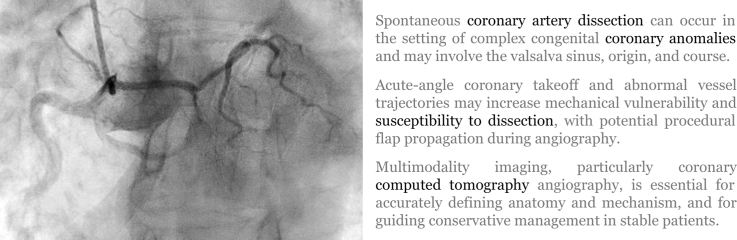

Spontaneous coronary artery dissection (SCAD) is a well-recognized nonatherosclerotic cause of acute coronary syndrome (ACS), particularly in women with few or no traditional cardiovascular risk factors (CVRFs). Although most cases involve a single coronary vessel and occur in otherwise typical coronary anatomy, increasing the use of advanced imaging has revealed important anatomical modifiers that may influence both disease expression and its management. Congenital coronary artery anomalies (CAAs) are rare but sometimes clinically relevant, especially when associated with high-risk anatomical features. The coexistence of SCAD and complex coronary anomalies has been infrequently reported and poses significant diagnostic and therapeutic challenges. We present a unique case illustrating the intersection of SCAD with a rare coronary tree origin from the right coronary sinus (RCS), underscoring the importance of multimodality imaging and conservative management.

**Take-Home Messages**

• Spontaneous coronary artery dissection can occur in the setting of complex congenital coronary anomalies and may involve the Valsalva sinus, origin, and course.
• Acute-angle coronary takeoff and abnormal vessel trajectories may increase mechanical vulnerability and susceptibility to dissection, with potential procedural flap propagation during angiography.
• Multimodality imaging, particularly coronary computed tomography angiography, is essential for accurately defining anatomy and mechanism and for guiding conservative management in stable patients.

**Visual Summary:**
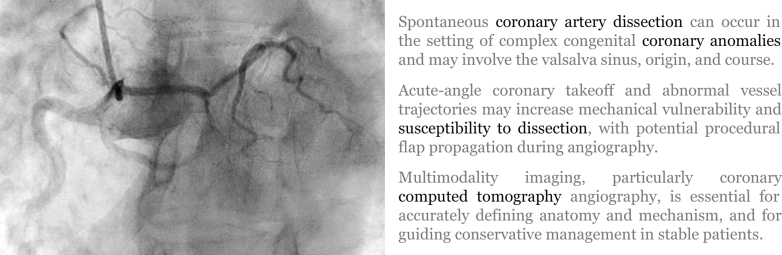
Spontaneous Coronary Dissection in Rare Coronary Anatomy

## Clinical Case

A 63-year-old woman with no known CVRFs presented with ACS. She reported no family history of cardiomyopathy, sudden death, or connective tissue disorders and had no history of exertional angina. Her past medical history included mild soft-tissue disorders (such as trigger fingers, spontaneous partial rotator cuff tear during light activity), with no definite cutaneous stigmata of connective tissue disease.

**Figure 1:**
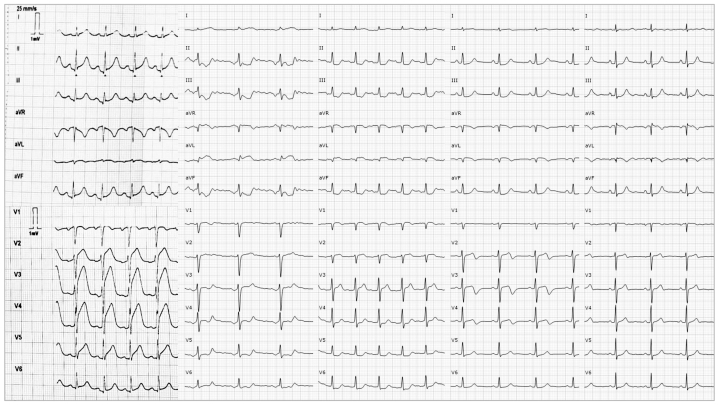
Serial Ischemic Electrographic Changes Evolution Shown from left to right: 1) Precatheterization hyperacute T waves and up to 8 mm of ST-segment elevation in V_2_ to V_5_ and 1 mm in aVL. 2) Immediately postcatheterization qR pattern and 1.5 mm of ST-segment elevation in aVL and downsloping ST straightening in V_3_ to V_5_, suggestive of active lateral ischemia. 3) Mild residual ST-segment elevation in leads V_1_ and V_2_ and aVL and ST straightening in V_5_ and V_6_ and the inferior leads 12 hours post catheterization. 4) Persistent anterolateral changes and development of biphasic T waves in V_2_ to V_4_ (Wellens type 1 pattern) 36 hours post catheterization, suggesting established anterolateral necrosis and reperfusion of septal and anterior territories (left anterior descending (LAD) territory). 5) Normalization of the remaining abnormalities 60 hours post catheterization, indicating localized necrosis in the left circumflex artery territory.

She described chest pain at rest without preceding exertion or any recalled Valsalva-related activities (eg, coughing). Home blood pressure was reported as 170/90 mm Hg, markedly above her usual readings. Electrocardiography showed signs of active ischemia in the septal, anterior, and lateral leads (Figure 1). Given persistent angina, she received a loading dose of dual antiplatelet therapy and was transferred for invasive coronary angiography.

**Figure 2:**
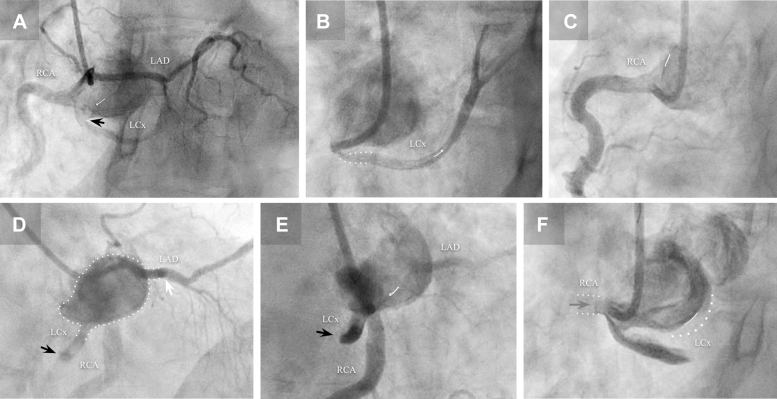
Common Coronary Sinus Origin of the Coronary Tree and Dissection Flap (A) Right coronary sinus (RCS) catheterization (LAO 31°, CAU 2.9° projection) showing the anterior LAD with an interarterial course. Within the same common coronary sinus, a dissection flap is visible (white arrow), together with a more lateral takeoff of a dominant and well-developed RCA located directly above left circumflex artery (LCx). (B) Subselective catheterization of the LCx from an acute-angle origin (LAO 42.2°, CAU 0.5° projection), demonstrating an intimal flap (white arrow) from its takeoff, propagated from the RCS. (C) Subselective RCA ostium contrast injection (LAO 29.9°, CAU 29.4° projection) showing its close proximity to the floating intimal flap within the RCS (white line). (D) Lateral (RAO 30°, CAU 6°) projection showing subselective catheterization of the LAD ostium, with contrast retained within the false lumen of the proximal dissected segment (white arrow), the LCx lumen (black arrow), and the RCS (white dots). (E) Lateral projection (RAO 26.8°, CRA 1.8°) showing RCS false lumen catheterization with an intimal flap progressing within it (white arrow), from the LAD to the LCx ostia, ultimately advancing and occluding it (black arrow). (F) Anterior projection (LAO 33.2°, CRA 0.7°) true lumen catheterization with an occluded RCA ostium due to catheter traction of the RCS intimal flap during selective catheterization (gray arrow); false lumen is hardly visualized at the Valsalva sinus (dotted lines).

**Figure 3:**
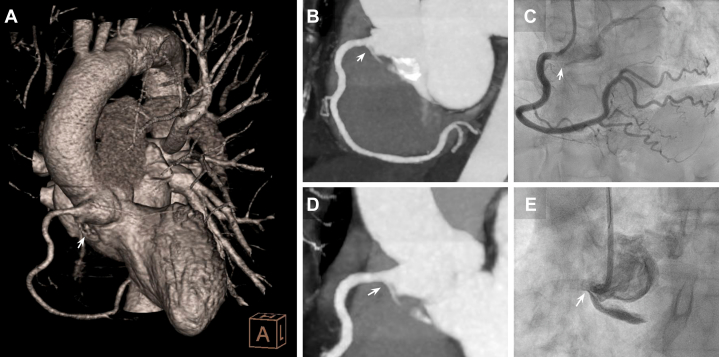
Comparison of Coronary Angiography Images With Arterial Phase Electrocardiographically Gated CCTA Reconstructions (A) CCTA 3D volume rendering showing the RCS origin of all 3 coronary arteries, specifically the retroaortic left circumflex artery (LCx (white arrow), dissected and with minimal flow. (B) Tomographic reconstruction in an anterior coronal plane demonstrating the intimal flap of the common coronary sinus progressing into the LCx ostium (white arrow), with angiographic contrast still retained in its false lumen. (C) Nonselective catheterization of the RCA ostium (LAO 8.1°, CRA 23.6° projection) showing the retroaortic LCx arising from its origin (white arrow). (D) Tomographic detail of the dissected LCx arising from the RCA ostium (white arrow). (E) Angiographic detail (LAO 33.2°, CRA 0.7° projection) of the RCS intimal flap progressing and occluding the proximal LCx lumen (white arrow), as well as the RCA ostium due to catheter mechanical traction.

**Figure 4:**
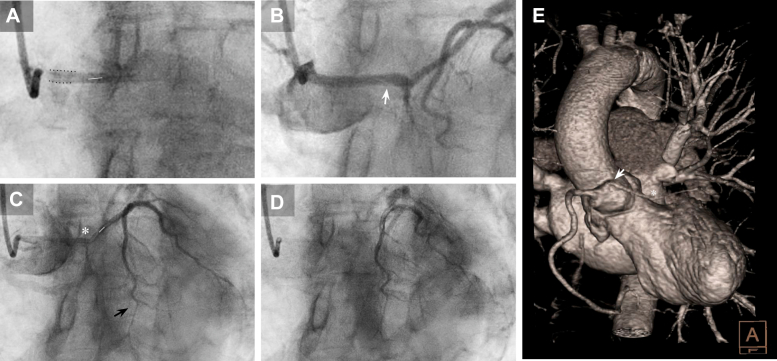
Angiographic and CCTA Comparison of the Dissected LAD (A) Anterior (LAO 31°, CAU 2.9°) projection detail of ostial catheter engagement of the LAD immediately after contrast injection, showing a subtle intimal flap (white line). (B) Detail (same projection) of the proximal LAD segment with an intimal flap (white line and arrow). (C) Complete angiographic LAD course (LAO 5.5°, CRA 41° projection) showing propagation of the intimal flap (white line) along its mid-to-distal segments (asterisk) and distal occlusion along an intraseptal trajectory (black arrow). (D) Late postcontrast injection sequence (same projection) demonstrating contrast retention within the false lumen of the mid-to-distal LAD. (E) CCTA 3D volume rendering in an anterior cranial view showing the LAD originating at an acute angle (white arrow).

Angiography revealed a dominant, well-developed right coronary artery (RCA) with no significant atheromatous disease and marked tortuosity. An anomalous origin of the entire coronary tree from the RCS was determined (Figure 2). Although difficult to visualize with nonselective catheterizations, the origin of a small, underdeveloped left circumflex artery (LCx) was identified, arising from the RCA ostium with a retroaortic course and initially TIMI 3 flow (Figures 2 and 3).The left anterior descending artery (LAD) appeared to have an interarterial course and features consistent with SCAD in its mid-to-distal segments, with TIMI 2 flow (Figure 4, Videos 1 and 2). Consequently, the anteroseptal ST elevation was attributed to the absence of distal flow to the apical territory. As the catheterization progressed, the patient reported improvement of angina and remained hemodynamically stable.

However, during one of the nonselective contrast injections, she reported chest pain recurrence and presented abrupt hypotension that required vasopressor support. Contrast retention was observed in the inferior portion of the RCS, initially suggestive of a possibly iatrogenic dissection flap that progressed into the LCx, obstructing its flow, and involved the RCA ostium due to catheter traction (Figures 2 and 3, Videos 3 and 5); LAD flow did not show any significant change during these events. Due to concerns about aortic root dissection, protamine sulfate was administered, and the procedure was terminated.

**Figure 5:**
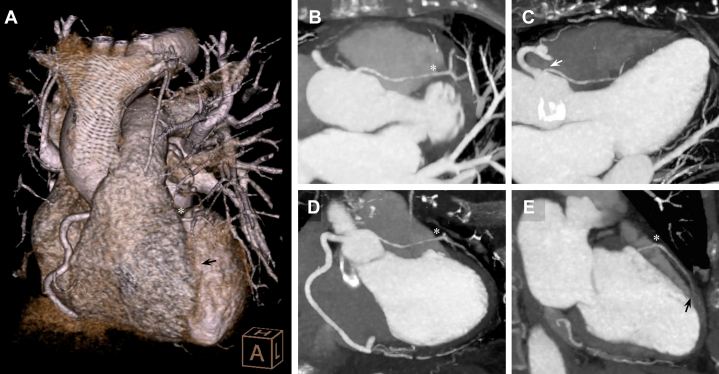
Multiplanar and Volumetric CCTA Reconstructions of the Ectopic LAD Course (A) CCTA 3D volume rendering in a left anterior oblique cranial view demonstrating a normal RCA anatomy and the LAD coursing posterior to the RVOT before emerging in the mid-anterior interventricular groove (black arrow). (B) Longitudinal cranial projection reconstruction of the LAD course, completing its posterior trajectory relative to the RVOT (asterisk) and highlighting luminal tapering in the context of proximal dissection and mid-intramyocardial course. (C) Longitudinal 3-chamber reconstruction showing the dissected proximal LAD course with its acute-angle origin in the RCS (white arrow). (D) LVOT oblique longitudinal reconstruction depicting the dissected LAD (asterisk). (E) Longitudinal 2-chamber reconstruction showing the mid-dissected LAD (asterisk) along the anterior interventricular course, with minimal distal contrast opacification (black arrow).

An emergent electrocardiographically gated contrast-enhanced computed tomography angiography (CCTA) confirmed a small dissection flap in the basal RCS. The RCA was adequately opacified, while the LAD closely matched the angiographic appearance with a tapered, partially opacified lumen suggestive of SCAD, probably from its origin (Figure 5). The retroaortic LCx showed an intimal dissecting flap, contrast retention without opacification, confirming its occlusion (Figure 3).

Upon admission to intensive care, the patient reported minimal residual chest discomfort, and her hemodynamics rapidly stabilized, allowing withdrawal of vasoactive support. The admission electrocardiogram demonstrated a shift in acute ischemia location (Figure 1), with normalization of the anteroseptal ST segments and new evident lateral ST elevation (aVL and I). Mechanical complications and significant ventricular dysfunction were excluded by bedside echocardiography. After careful risk-benefit assessment, given clinical and hemodynamic stability, evidence of SCAD, and a possibly iatrogenic RCS dissection, a conservative management strategy was adopted.

**Figure 6:**
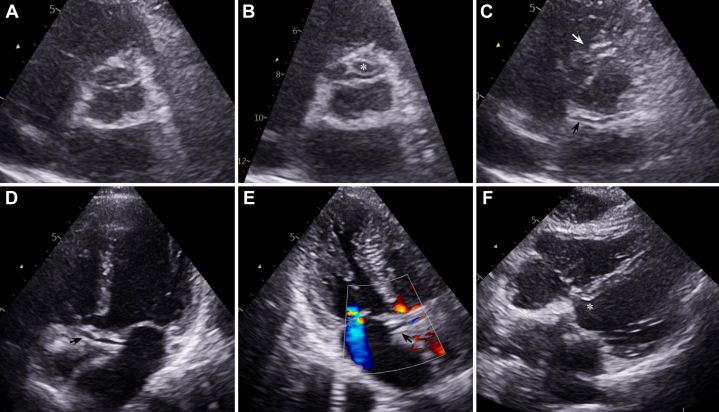
Origin and Course of Anomalous Coronary Arteries on TTE (A) Parasternal short-axis (PSAX) view showing a tricuspid aortic valve with normal characteristics. (B) Slightly higher PSAX view showing an intimal dissection flap in the RCS (asterisk) and the LAD origin. (C) Supravalvular PSAX view showing the posterior course of the LAD (white arrow) and the retroaortic course of the LCx (black arrow). (D) Modified apical 5-chamber view demonstrating the “RAC sign” (retroaortic anomalous coronary), with the LCx (black arrow). (E) Apical 3-chamber view with color Doppler showing the retroaortic LCx with minimal internal flow (black arrow). (F) Subcostal 4-chamber view showing the intramyocardial anteroseptal course and angulation at the onset of the mid-LAD (asterisk).

Serial electrocardiographic evolution (Figure 1) confirmed the LAD territory reperfusion, together with localized necrosis in the LCx territory. Peak high-sensitivity troponin T reached 3,760 ng/L at 72 hours. Transthoracic echocardiography demonstrated a mildly reduced ejection fraction with regional systolic dysfunction involving territories corresponding to the LCx and distal LAD, preserved right ventricular function, and an estimated pulmonary arterial systolic pressure of 50 mm Hg in the setting of elevated left-sided filling pressures, both of which subsequently normalized several days after the event. The RCS intimal flap and anomalous coronary course could also be identified (Figure 6, Videos 7 to 9).

**Figure 7:**
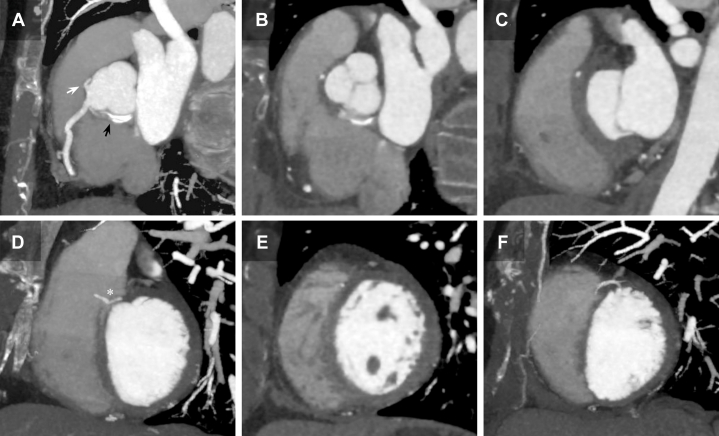
CCTA Axial-Plane Reconstruction of the LAD Course (A) Supravalvular aortic plane showing the origin of all 3 coronary arteries from the RCS: the LCx with the dissection flap (black arrow) and retained angiographic contrast, and the LAD originating at an acute-angle (white arrow). (B) Aortic valvular plane showing the LAD course. (C) Mitral valvular plane with the LAD traversing the anterior interventricular septum. (D) Basal-segment plane showing the onset of the significantly angulated mid-LAD segment immediately after the first septal branch. (E) Midsegment plane with poorly opacified intramyocardial mid-LAD lumen. (F) Apical-segment plane showing re-emergence of the distal LAD in its epicardial course.

CCTA multiplanar reconstructions subsequently allowed a more detailed characterization of the LAD origin and course (Figures 5 and 7), highlighting its acute-angle takeoff, close relationship with the right ventricular outflow tract, and intramyocardial septal trajectory in the mid-to-distal segments. A more focused review of the angiographic footage revealed an initial sinusal dissection involving the LAD and LCx ostia (Figure 4). This observation prompted reconsideration of the RCS dissection as potentially spontaneous rather than purely iatrogenic, even considering the possibility that it originated at the acutely angled LAD or LCx takeoffs and extended both distally and proximally into the RCS, eventually leading to LCx occlusion during catheterization. The transient RCA occlusion was attributed to catheter-mediated flap traction, retracting into the RCS with restoration of normal coronary flow after its withdrawal, as evidenced by CCTA (Figure 3).

The patient showed a favorable clinical evolution, with complete resolution of angina and no post-ACS complications. Conservative medical management of SCAD was pursued. A comprehensive etiological workup, including screening for inherited connective tissue disorders, autoimmune arteritis, and fibromuscular dysplasia, was negative. She was discharged on acetylsalicylic acid, bisoprolol, and statins following a newly diagnosed dyslipidemia. At 3 months, a follow-up coronary CT angiography was performed, showing no changes, and a stress echocardiogram was negative for ischemia. At 6 months of follow-up, the patient remains asymptomatic.

## Discussion

SCAD is a nonatherosclerotic, nontraumatic ACS cause that predominantly affects women and often occurs in patients with few traditional CVRFs. It is estimated to account for approximately 1% to 4% of ACS presentations and for up to one-third in women younger than 50 years, most commonly involving a single coronary vessel, typically the mid-to-distal segments, with the LAD being the most frequently affected artery.^1^, ^2^, ^3^ Large observational series have highlighted its strong association with fibromuscular dysplasia and other systemic arteriopathies, as well as frequent preceding emotional or physical stressors.^1^^,^^2^ A mid-term recurrence risk of up to 10% has been reported in several studies at a median follow-up of 3 years.^1^^,^^2^^,^^4^

CAAs constitute a heterogeneous group of congenital abnormalities involving coronary origin and course, with an overall prevalence of 0.3% to 1% in angiographic series.^5^^,^^6^ Within this spectrum, the origin of all 3 epicardial coronary arteries from the RCS, most often through a single ostium, is exceptionally rare, with an estimated incidence of 0.008%.^5^^,^^7^^,^^8^ Although many of these variants may remain clinically silent, specific anatomic features, including an acute-angle takeoff, retroaortic, interarterial, intramural, or intraseptal courses have been associated with myocardial ischemia, malignant arrhythmias, or sudden cardiac death.^5^, ^6^, ^7^

The coexistence of SCAD and complex CAAs has been rarely reported. Yang et al^8^ described a woman with multivessel SCAD, in whom all 3 coronary arteries arose from a common origin in the RCS, and conservative management was favored due to distal vessel involvement and technical interventional challenges, closely paralleling the anatomical complexity and therapeutic considerations of the present case.

This case adds to the limited evidence linking SCAD with complex CAAs. The finding of a coronary sinus dissection may support the hypothesis of iatrogenic dissection. However, previous electrocardiographic abnormalities and initial subselective sequences with a flap extension from vulnerable, acutely angulated coronary takeoffs suggest spontaneous dissection, although spontaneous dissection of the anterior descending artery with concomitant iatrogenic dissection of the sinus and involvement of the left coronary artery prior to the first contrast injection cannot be categorically ruled out. This scenario highlights the need for heightened procedural caution in patients with complex CAAs, as subsequent procedural manipulation undoubtedly contributed to flap traction and coronary flow compromise. As suggested by isolated reports in the setting of CAAs, this pattern highlights how an intrinsically vulnerable vessel wall, together with a mechanically disadvantaged coronary origin and abnormal coronary geometry, may contribute to increased susceptibility to SCAD and potential procedural propagation.^9^, ^10^, ^11^ In this context, multimodality imaging, particularly coronary CCTA, plays a central role in accurately defining coronary origin, course, and high-risk features that may be incompletely appreciated by conventional angiography.^5^^,^^7^

Current expert consensus supports conservative management of SCAD when coronary flow is preserved, and hemodynamic stability is maintained, especially given the heightened procedural risks associated with CAAs.^1^, ^2^, ^3^^,^^12^ Single antiplatelet and beta-blocker therapy are commonly employed in this setting, largely based on observational data and expert opinion, with limited direct evidence supporting their efficacy for SCAD-specific outcomes.^1^, ^2^, ^3^ Although dual antiplatelet therapy loading was initially administered, its role in SCAD prior to coronary angiography is controversial and has progressively had a lower level of recommendation due to its potential harmful effects and hemorrhagic complications. In fact, in SCAD, it may lead to further extension of the dissection and intramural hematoma propagation, with no proven clinical benefit^1^, ^2^, ^3^; however, in this case, it did not significantly influence management. Beyond the acute event, patients require follow-up according to established SCAD recommendations, while complex CAAs warrant individualized ischemic and arrhythmic risk stratification.^1^, ^2^, ^3^^,^^5^ In this case, long-term outcomes and recurrence risk remain uncertain given the complex anatomy and the currently limited follow-up.

## Conclusions

This case illustrates a rare but clinically meaningful intersection between SCAD and complex CAAs. High-risk anatomical features can confer mechanical coronary vulnerability that could predispose to dissection, with potential exacerbation during coronary instrumentation. Recognition of this anatomy and its clinical implications, supported by a careful multimodality imaging approach, is critical to accurately define the underlying mechanism and avoid an unnecessary and potentially harmful intervention. In the setting of SCAD, conservative management is preferred when coronary flow is preserved and hemodynamic stability is maintained. Although uncommon, complex CAAs carry important diagnostic and therapeutic implications and require individualized risk stratification to guide long-term management.

## Funding Support and Author Disclosures

The publication of this paper has been possible thanks to the financial support of the Interhospital Foundation for Cardiovascular Research (FIC, Madrid, Spain). The authors have reported that they have no relationships relevant to the contents of this paper to disclose.

## Appendix

Video 1 First Angiographic Sequence (LAO 5.5°, CRA 41°) Demonstrating LAD Course With Intimal Flap Propagation Along its Mid-to-Distal Segments (With TIMI 2 Flow and Parietal Contrast Retention Within the False Lumen), as Well as Distal Occlusion Along an Intraseptal Trajectory On careful review of the subselective catheterization, the courses of the RCA and LCx can be identified, with a possible dissection flap extending from the RCS into the LCx.

Video 2 Second Angiographic Sequence (LAO 5.5°, CRA 41° Projection) With Intimal Flap Propagating Along Mid-to-Distal LAD Segments, Distally Occluded

Video 3 Third Angiographic Sequence (LAO 42.2°, CAU 0.5°), Sub-Selective Catheterization of the Retroaortic LCx From an Acute-Angle Origin Demonstrating an Intimal Flap From its Takeoff, Propagated From the RCS

Video 4 Subsequent Lateral Angiographic Sequence (RAO 26.8°, CRA 1.8°) Immediately after LCx catheterization, opacification of the RCS false lumen was observed, with an intimal flap extending from the LAD to the LCx ostia, resulting in proximal LCx occlusion and contrast retention.

Video 5 Anterior Angiographic Projection (LAO 33.2°, CRA 0.7°) Demonstrating RCS True Lumen Catheterization With an Occluded LCx Ostium, a Jet of Contrast Leakage Into the RCS Suggestive of an Intimal Flap Tear in the Context Of Recent Challenging Catheterization, as well as an Occluded RCA Ostium due to Catheter Traction of the RCS Intimal Flap During Selective Catheterization

Video 6 Transthoracic Echocardiography Parasternal Short-Axis Supravalvular Aortic View Demonstrating the Posterior Course of the LAD Relative to the RVOT and the Retroaortic Course of the LCx

Video 7 Transthoracic Echocardiography Apical 4-Chamber View Demonstrating a Retroaortic LCx (“RAC Sign”)

Video 8 Transthoracic Echocardiography Apical 2-Chamber View Demonstrating Regional Wall Motion Abnormalities Involving the Basal Anterior Segment and the Apical Cap

Video 9 Transthoracic Echocardiography Apical 3-Chamber View Demonstrating the Retroaortic Course of the LCx, Mild Mitral Regurgitation, and Regional Wall Motion Abnormalities Involving the Basal Anterolateral and Apical Septal Segments
